# Recent Trends in Pharmaceutical Analytical Chemistry

**DOI:** 10.3390/molecules25163560

**Published:** 2020-08-05

**Authors:** Constantinos K. Zacharis, Catherine K. Markopoulou

**Affiliations:** Laboratory of Pharmaceutical Analysis, Department of Pharmaceutical Technology, School of Pharmacy, Aristotle University of Thessaloniki, 54124 Thessaloniki, Greece; amarkopo@pharm.auth.gr

Modern analytical chemistry plays a vital role in pharmaceutical sciences. It provides precise and accurate data supporting the processes related to drug discovery and development. Some examples include the purity of drug substances during synthesis, pharmacokinetic studies, drug stability, elucidation of the drug metabolic pathways, drug–protein interactions, etc. New methodologies, state-of-the art instrumentation and materials, and automated systems—offering precise and accurate analytical data—have become essential prerequisites in this field. Moreover, chemometrics have been proved to be a beneficial tool for the optimization of method parameters and can also identify and minimize the sources of variability that may lead to poor method robustness.

The present Special Issue comprises twelve full-length research articles covering the latest research trends and applications of pharmaceutical analytical chemistry. An interesting cell-based bioassay was proposed by the research group of C. Rao and J. Wang for the determination of the bioactivity of long-acting growth hormone as a potential alternative biopharmaceutical for the treatment of growth hormone deficiency [1]. The method is based on the usage of a luciferase reporter gene system, which is involved in the full-length human GH receptor (hGHR) and the SIE and GAS (SG) response element. The proposed protocol can be used in routine analysis and it was validated according to ICH guidelines and the Chinese Pharmacopoeia.

The research group of X.-S. Feng presented “*Pharmacokinetics and Tissue Distribution of Alnustone in Rats after Intravenous Administration by Liquid Chromatography-Mass Spectrometry*” [2]. A simple and fast LC-MS/MS method was developed to evaluate the pharmacokinetic and tissue distribution profiles of alnustone in rats. The sample preparation involved simple protein precipitation using acetonitrile. After the optimization of LC-MS/MS conditions, the method was fully validated according to the bioanalytical method validation guidance for industry (FDA). The authors concluded that alnustone is predominantly distributed in the lung and liver tissues within 1 h and therefore these tissues might be the target organs for the curative effect of the drug. Analogous research has been published by the same authors related to the “*Pharmacokinetics and Tissue Distribution of Anwuligan in Rats after Intravenous and Intragastric Administration by Liquid Chromatography-Mass Spectrometry*” [3]. In contrast to their previous work [2], a liquid–liquid extraction-based sample preparation protocol was developed and optimized for the isolation of anwuligan from rat plasma and tissues with minimum matrix effects. According to their findings, the half-time elimination of the drug was relatively shorter after intragastric administration compared to the intravenous one. An interesting investigation was also published by the same researchers in their publication “*Interaction Effects between Doxorubicin and Hernandezine on the Pharmacokinetics by Liquid Chromatography Coupled with Mass Spectrometry*” [4]. One of the primary targets of the work was the development of an LC-MS/MS method for the determination of both drugs in rat plasma and also to compare their pharmacokinetic data after intravenous administration in rats. According to their findings, there were significant differences in the pharmacokinetic profiles—especially in the C_max_ and AUC_0-∞_ parameters of doxorubicin—indicating that hernandezine could improve the absorption of the anticancer drug. The outcome of this research might be used as clinical guidance for doxorubicin and hernandezine to prevent adverse reactions.

Dr. El-Nahhas et al., in their interesting contribution “*A Hydrophilic Interaction Liquid Chromatography–Tandem Mass Spectrometry Quantitative Method for Determination of Baricitinib in Plasma, and Its Application in a Pharmacokinetic Study in Rats*”, presented quantitation data from the determination of baricitinib in rat plasma [5]. A liquid–liquid extraction procedure with two extraction solvents (n-hexane and dichloromethane) was proposed for drug isolation from biological matrices, avoiding the potential enhancement or suppression of the MS signal. The authors investigated the separation of baricitinib and the internal standard under HILIC conditions, achieving a 3-min isocratic separation.

A sensitive UHPLC-MS/MS method has been published by F.-H. Meng et al. in their interesting article entitled “*Development and Validation of a Sensitive UHPLC-MS/MS Method for the Measurement of Gardneramine in Rat Plasma and Tissues and Its Application to Pharmacokinetics and Tissue Distribution Study*” [6]. Gardneramine is a monoterpenoid indole alkaloid with an exceptional central depressive effect and actions on myocardium. The pharmacokinetic properties and tissue distribution of the selected drug have been thoroughly investigated after its intravenous administration. To support this task, the authors developed a fast UHPLC-MS/MS method in combination with simple protein precipitation for sample preparation.

Pharmacokinetic profiling data of thiopurine immunosuppressants and folic acid were provided by the research group of Mornar in the report entitled “*Pharmacokinetic Profiling and Simultaneous Determination of Thiopurine Immunosuppressants and Folic Acid by Chromatographic Methods*” [7]. The authors evaluated the pharmacokinetic profiling of azathioprine, 6-mercaptopurine, 6-thioguanine, and folic acid using various chromatographic techniques and in silico methodology. An HPLC method was also developed for the simultaneous determination of the analytes in their commercially available formulations.

An automated stopped-flow fluorimetric sensor based on zone fluidics was proposed by P. D. Tzanavaras et al. in their work “*Automated Stopped-Flow Fluorimetric Sensor for Biologically Active Adamantane Derivatives Based on Zone Fluidics*” [8]. One of the main scopes of the research was to develop an automated method for the fast determination of amantadine, memantine, and rimantadine for the quality control of their commercially available formulations. Interestingly, an amantadine-containing formulation obtained from a third—non-EU—country was found to be out of specification. These results were confirmed using a validated HPLC method. 

Dr. Masadome and co-workers, in their article “*Determination of Polyhexamethylene Biguanide Hydrochloride Using a Lactone-Rhodamine B-Based Fluorescence Optode*”, reported a new approach for the quantitation of polyhexamethylene biguanide using a lactone–rhodamine B fluorescence optode [9]. The analyte is a cationic polyelectrolyte which is used for disinfectants in contact lens detergents and sanitizers for swimming pools. The principle of the method is based on the fluorescence quenching of a lactone–rhodamine B-containing optode membrane caused by the analyte. The method is quite simple without using toxic reagents.

A high-throughput bar adsorptive microextraction for the analysis of ketamine and norketamine in urine has been proposed by J.M.F. Nogueira et al. in their interesting publication “*A Fast and Validated High Throughput Bar Adsorptive Microextraction (HT-BAμE) Method for the Determination of Ketamine and Norketamine in Urine Samples*” [10]. The identification and quantification of both drugs were performed using large-volume injection gas chromatography–mass spectrometry operating in the selected ion monitoring mode. A set of parameters that could influence the performance of the microextraction was investigated and optimized. The proposed analytical scheme has the possibility of performing parallel microextractions and the subsequent desorption of up to 100 samples in a single apparatus in just 45 min.

Dr. Markopoulou et al., in their study “*Partial Least Square Model (PLS) as a Tool to Predict the Diffusion of Steroids across Artificial Membranes*”, presented data regarding the ability of a drug to permeate human tissues using a partial least square-based statistical approach [11]. The researchers attempted to decode the drug permeability by correlating the apparent permeability coefficient of 33 steroids with their physicochemical and structural properties. The apparent permeability coefficient of the compounds was determined by in vitro experiments. The obtained models could be utilized to predict the permeability of a new drug candidate without animal experiments. 

Last, but not least, Drs. Balayssac and Gilard and their colleagues in the publication “*Chemometric Analysis of Low-field 1H NMR Spectra for Unveiling Adulteration of Slimming Dietary Supplements by Pharmaceutical Compounds*” presented the development of a novel chemometric-based NMR approach for unveiling the adulteration of slimming dietary supplements [12]. The authors analyzed 66 dietary supplements using low-field ^1^H-NMR to build the PLS model. The potential limitations of the proposed method consist of the poor sensitivity and the low spectral resolution of the low-field ^1^H-NMR instrument.

As Guest Editors of this Special Issue, we are sincerely grateful to all the contributors for their outstanding cooperation and valuable support. Special thanks are also due to all the peer reviewers for their valuable suggestions and criticisms.
